# Mild-to-wild plasticity of Earth’s upper mantle

**DOI:** 10.1038/s41561-026-01920-7

**Published:** 2026-02-09

**Authors:** David Wallis, Kathryn M. Kumamoto, Thomas Breithaupt

**Affiliations:** 1 Department of Earth Sciences, University of Cambridge, Cambridge, UK; 2 Lawrence Livermore National Laboratory, Livermore, CA USA

**Keywords:** Structural geology, Mineralogy, Geodynamics

## Abstract

Flow of Earth’s upper mantle has long been considered to occur by slow, near-continuous creep. This behaviour is observed in classical high-temperature deformation experiments and is a fundamental component of geodynamic models. The latest generation of high-resolution experiments, however, have revealed that materials ranging from metals to ice exhibit a spectrum of behaviours, termed mild-to-wild plasticity, that range from this mild continuous flow to intermittent wild fluctuations in plastic strain rate. Here we show, using nanoindentation experiments, that olivine exhibits measurable wildness, even under conditions at which its behaviour is expected to be relatively mild. Specifically, during experiments on olivine single crystals at room temperature, continuous plastic flow is punctuated by intermittent bursts of displacement with log-normally distributed magnitudes, indicating avalanches of correlated dislocation motion that account for ~8 ± 6% of the plastic strain. The framework of mild-to-wild plasticity predicts that wildness should increase with depth in Earth, with flow of the asthenosphere occurring almost entirely by wild fluctuations of deformation at the grain scale. The recognition of intermittent plasticity in geological materials provides additional constraints on models of dislocation-mediated flow and raises questions about the mechanisms of transient instabilities in otherwise ductile regimes, such as deep earthquakes and slow-slip events.

## Abstract (web summary)

Flow of Earth’s upper mantle may include intermittent wild fluctuations in plastic strain rate of increasing frequency with depth, according to results of nanoindentation experiments on olivine crystals.

## Main

The flow of hot but solid rocks in Earth’s mantle is an archetypal slow and steady process. The motion of Earth’s surface appears smooth and continuous when observed by geodetic techniques^1^, implying that the flow of hot, ductile rocks at the bases and margins of plates is similarly steady, at least when observed at large spatiotemporal scales. Intermittent bursts of deformation do occur as earthquakes, mostly in the cold, brittle upper crust, yet the viscous deformation of deeper, hotter rocks that is induced in the aftermath of earthquakes also appears as smooth flow when detected at the surface^2,3^. These observations are mirrored in classical plasticity and creep experiments, in which millimetre- or centimetre-sized crystals flow steadily^4–6^. However, it cannot be taken for granted that these observations accurately characterize the nature of flow at and below the grain scale in the upper mantle, nor that they provide a complete picture of the characteristics of flow at larger scales. For instance, some sets of geophysical observations are postulated to result from transient instabilities of deformation under conditions typically associated with viscous flow. For example, some earthquakes in subduction zones occur at depths where pressure is expected to suppress frictional failure and instead have been hypothesized to result from thermal runaway during viscous flow^7,8^. Similarly, slow-slip events have also been suggested to occur due to transient instabilities of viscous flow resulting from accelerated dislocation glide^9^. These observations motivate a reexamination of the fundamental nature of viscoplastic flow in geological materials.

Recent developments in the material sciences have revealed a spectrum of deformation behaviours termed mild-to-wild plasticity^10–12^. Advances in techniques with high spatial and/or temporal resolution, including acoustic-emission monitoring^10–14^, micropillar deformation^15,16^ and nanoindentation^15,17,18^, make it possible to probe the collective dynamics of populations of dislocations during plasticity and creep. In some cases, flow appears smoothly continuous, even at small length scales, and is termed mild plasticity^10–12^. Often, however, deformation occurs in discrete bursts, implying the spatiotemporally correlated motion of groups of dislocations in avalanches, termed wild plasticity^10–12^.

Whether a material exhibits mild or wild plasticity depends systematically on the length scale of observation and the lattice resistance to dislocation glide at a given glide velocity and temperature^11,12^. Over large length scales, discrete dislocation motion is difficult to detect and an averaged mild behaviour is observed. At smaller length scales, bursts of dislocation motion are easier to detect and increasingly dominate the observed behaviour. Likewise, when lattice resistance is high, elastic interactions due to the motion of one dislocation have little impact on neighbouring dislocations that are effectively held in place. However, when lattice resistance is low, the motion of one dislocation can trigger an avalanche of dislocations in its vicinity.

These effects have important implications for the nature of plasticity and creep in the upper mantle, where extrapolation of laboratory flow laws^19^ and observations of seismic anisotropy^20^ indicate that deformation occurs predominantly by dislocation-mediated mechanisms. Whereas deformation of the mantle appears mild when observed by geodetic techniques^1–3^ over length scales of 10^3^–10^6^ m, plasticity and creep at smaller scales may be wilder. Furthermore, although classical experiments do probe smaller length scales on the order of 10^−3^–10^−2^ m, at which plasticity again appears mild^4–6^, the difference in strain rates between deformation in the laboratory and in nature generates large differences in effective lattice resistance between the two settings. A recently calibrated flow law for dislocation glide in olivine at high temperatures predicts that lattice resistance under the high temperatures and low strain rates of the asthenosphere will be dramatically less than in experiments^21^, increasing the potential for wild plasticity. Finally, the orthorhombic symmetry and pronounced viscoplastic anisotropy of olivine limits the number of active slip systems, thereby reducing the potential for short-range dislocation interactions^22^ that would increase the glide resistance and promote mild plasticity^11,12^. Instead, long-range elastic interactions among dislocations dominate during plasticity^6,23^ and creep^21,24,25^ of olivine, again increasing the potential for wild plasticity^11,12^. These considerations suggest that the fundamental nature of plasticity and creep in the upper mantle may be different to intuition gained from observations at large length scales or laboratory strain rates. Instead, the deformation of olivine should be evaluated within the framework of mild-to-wild plasticity to assess the underlying nature of collective dislocation dynamics in various contexts. This reappraisal will provide new constraints for rheological models based on dislocation motion, and will provide a first step towards assessing the extent to which macroscopic instabilities during ductile flow, such as deep earthquakes and slow-slip events, may be linked to intermittent behaviours at smaller length scales.

In this Article we analyse the deformation of olivine at the smallest length scales available in the laboratory, using instrumented nanoindentation^26^ to test whether intermittent plasticity occurs. These experiments reveal the occurrence of dislocation avalanches and allow olivine to be placed on the spectrum of mild-to-wild plasticity. This result allows the framework of mild-to-wild plasticity to make predictions of changes in the degree of wildness as the lattice resistance is extrapolated to natural conditions. This analysis suggests that plasticity of the lithosphere is mild across most length scales. However, creep of the asthenosphere is probably wild, at least up to the grain scale, and potentially beyond. These predictions constitute a previously unrecognized mild-to-wild transition with depth in Earth’s upper mantle.

## Dislocation avalanches in nanoindentation experiments

**Fig. 1:**
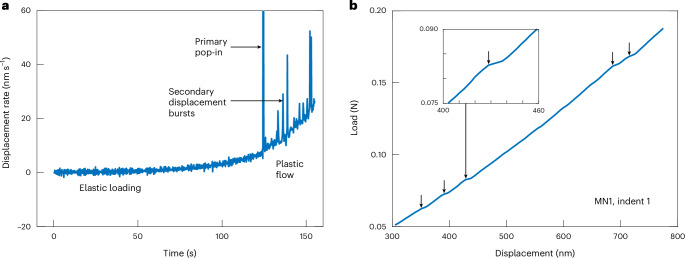
Displacement bursts during plastic flow. **a**, Typical time series of displacement rate, indicating elastic loading and plastic flow separated by the primary pop-in, along with secondary displacement bursts during plastic flow. **b**, Typical load–displacement curve from the plastic-flow portion of the test after the primary pop-in. Black arrows indicate secondary displacement bursts that are detected and included in analysis (sample MN1, indent 1). Inset: enlargement of the third displacement burst.

Nanoindentation tests using a spheroconical indenter tip on single crystals of olivine with low defect densities exhibit three characteristic portions of the load–displacement curve and corresponding pseudo-stress–strain curve^26^, as indicated in Fig. 1a. Initial loading is elastic and follows Hertzian contact mechanics. Elastic loading ends in a displacement burst, termed a ‘pop-in’, which marks the onset of plastic flow. Subsequent deformation proceeds by plastic flow and exhibits strain hardening. Both dislocations and microcracks are present in the deformed zone after each test^23^. However, both theoretical considerations and observational evidence indicate that, when indented by a tip with a radius on the order of a few micrometres or less, only dislocation motion occurs during loading, whereas microcracks initiate during unloading, even in brittle ceramics^26,27^. In olivine, the initial pop-in has been studied in detail to analyse dislocation nucleation and the onset of dislocation motion^26^. We instead focus on the third portion of the test to investigate dislocation dynamics during ongoing plastic flow.

Our key observation is that, after the initial pop-ins, the load–displacement curves exhibit smooth, continuous plastic flow punctuated by intermittent secondary displacement bursts. These bursts are evident as peaks in displacement rate in a typical test (Fig. 1a). Figure 1b presents the plastic-flow portion of the same test, with the displacement bursts identified by our detection routine marked by black arrows. Segments of continuous flow proceed over displacements on the order of tens to hundreds of nanometres. In contrast, the magnitudes of displacement bursts have a mean of 5.4 nm, standard deviation (s.d.) of 2.9 nm and maximum of 25.6 nm, but typically occur within one sampling time interval of 0.2 s. Displacement bursts were detected in 83% of tests, and between 40 and 103 displacement bursts were detected in total for each sample.

**Fig. 2:**
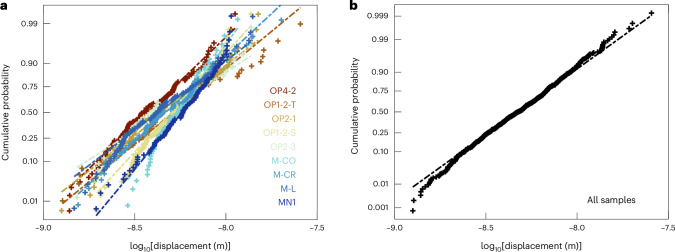
Probability distributions of displacement bursts. **a**, Normal probability plot of the logarithm of magnitudes of secondary displacement bursts in each sample. The vertical axis is scaled such that a normal distribution plots as a straight line. As we take the logarithm of displacement, datasets that plot as a straight line are log-normally distributed. Dashed lines indicate straight lines fitted to the distributions from each sample. Each point represents the cumulative probability associated with one bin of log displacement. **b**, Normal probability plot of the logarithm of magnitudes of secondary displacement bursts in all samples.

Analysis of the probability distributions of the magnitudes of displacement bursts provides additional insight into their characteristics. Figure 2 presents normal probability plots, in which the cumulative-probability axis is scaled such that a normal distribution falls on a straight line. We plot the logarithm of the magnitudes of displacement occurring during bursts so that a straight line indicates a log-normal distribution. Figure 2a shows that the distributions from all samples are close to straight lines. This overall behaviour is clear in Fig. 2b, in which the 695 displacement bursts across all samples are combined. The magnitudes of displacement bursts are log-normally distributed across an approximate range of 2–20 nm.

Similar log-normal distributions of secondary displacement bursts have recently been observed during spherical nanoindentation of Cu^17^. Cu does not fracture during nanoindentation with either spheroconical^28,29^ or sharp^30,31^ tips, providing further confirmation that secondary displacement bursts with these characteristics are formed by dislocation motion alone. In spheroconical nanoindentation, scale-invariant power-law distributions of displacement bursts, typical of correlated dislocation motion in many test systems^10–12,15,18,32^, do not emerge because the size of the indent and deforming volume beneath it, as well as the number and density of dislocations, and hence intensity of interactions among them, are evolving continuously throughout the test and thereby modulating the distribution of sizes of displacement bursts^17^. The complexity of these confounding effects precludes attempts to isolate or correct for them. However, the absence of this behaviour does not imply that the dislocation motion was mild, in which case Gaussian statistics would emerge^17^. Instead, the log-normal distribution of displacement-burst magnitudes is a signature of correlated dislocation motion in spheroconical nanoindentation^17^, with displacement bursts of 2–20 nm in olivine resulting from avalanches of up to approximately several tens of dislocations.

A key advantage of spherical nanoindentation is the capability to determine pseudo-stress–strain curves from load–displacement curves^33^ (Methods), allowing estimates of plastic strain accommodated during secondary displacement bursts. Across all samples, the mean plastic strain accommodated during each displacement burst is estimated to be 1.3 × 10^−3^, with s.d. of 1.1 × 10^−3^ and a maximum of 1.0 × 10^−2^. The fraction of the total plastic strain after the initial pop-in that is accommodated in secondary displacement bursts provides a quantitative estimate of wildness. This fraction varies between 4% and 12% among samples, with an average wildness of 8 ± 6% (1 s.d.) across all tests.

**Fig. 3:**
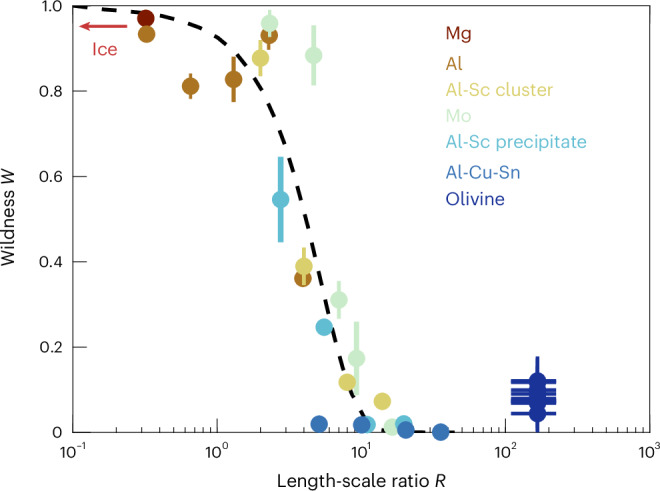
Wildness against the dimensionless length-scale ratio. Wildness is the fraction of plastic strain occurring in secondary displacement bursts. Uncertainties in wildness are plotted as the s.d. of wildness across different nanoindents made on each sample. The length-scale ratio *R* is computed using a shear modulus of 70 ± 5 GPa, a Burgers vector of 0.6 ± 0.1 nm and a lattice resistance of 1.4 ± 0.3 GPa (ref. ^6^). The uncertainties on these parameters are combined in quadrature to estimate the uncertainty in *R*. Data for metals are from refs. ^10,11^ and data for olivine are from this study. Figure adapted from ref. ^11^ under a Creative Commons license CC BY 4.0.

The framework of mild-to-wild plasticity allows comparisons of the wildness of different materials. In general, wildness varies as a function of the ratio *R* of length scales, *R* = *L*/*l*, where *L* is the system size and *l* is an internal length scale^11,12^. This internal length scale quantifies the relative resistance to dislocation motion from long-range elastic dislocation interactions and short-range obstacles through *l* = *Gb*/*τ*, where *G* is the shear modulus, *b* is the magnitude of the Burgers vector, and *τ* is the combined resistance imposed by the lattice, short-range dislocation interactions, solution hardening and/or precipitates^11,12^. In general, when *R* is large (that is, when the system is large, or strengthening from short-range obstacles dominates), plasticity appears mild, whereas when *R* is small (that is, when the system is small, or long-range elastic interactions dominate), plasticity appears wild^11,12^. Figure 3 presents this trend for several different materials.

The plasticity of olivine can also be considered in this context. We estimate the diameter of the deforming region beneath the indenter tip to be ~5 μm based on transmission electron micrographs^23^. Detailed experimental^6,21^ and microstructural^23,24,34^ analyses suggest that strain hardening in olivine is dominated by long-range elastic interactions, with negligible contributions from short-range interactions, solution strengthening or precipitates. We thus estimate *τ* to be a lattice resistance at room temperature, as half the differential stress predicted by a flow law for low-temperature plasticity that allows separation of the strength contributions from lattice resistance and hardening, giving a value of 1.4 GPa (ref. ^6^). These estimates of the size of the deforming region and the lattice resistance give *R* = 167. This high value of *R* and low values of wildness for olivine at room temperature are broadly consistent with the trend defined by other materials in Fig. 3. Although the wildness values of 4–12% estimated for olivine are slightly greater than those of, for example, Al–Cu–Sn measured at lesser values of *R*, they are low in the context of the full range of wildness across materials and represent only a small component of wildness amongst mild behaviour overall. Moreover, the range of wildness values estimated for olivine, and their difference from zero, are less than the scatter of ~20% wildness evident in the data for other materials at a given value of *R*, indicating that the estimates from olivine are broadly consistent with the overall trend.

## Mild-to-wild plasticity in the upper mantle

**Fig. 4:**
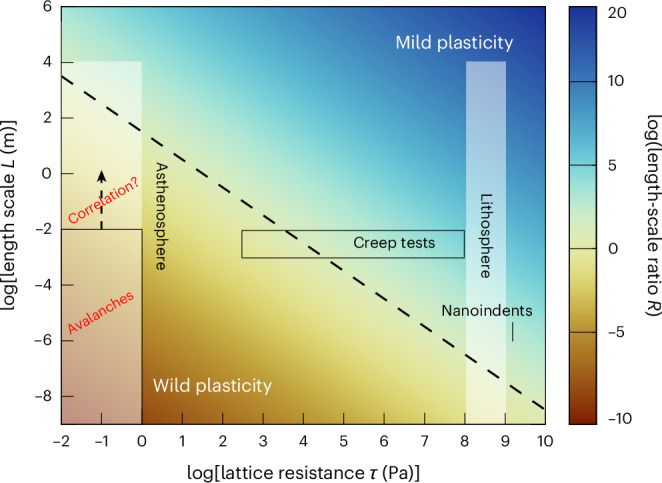
Predicted regimes of mild and wild plasticity. The dimensionless ratio *R* between the length scale of observation (*L*) and characteristic microstructural length scale (*l*) as a function of the length scale of observation and the lattice resistance to dislocation glide (*τ*). The length scale and lattice resistance of the nanoindentation experiments were estimated from ref. ^23^ and ref. ^6^, respectively. The approximate range of lattice resistances during high-temperature creep tests was estimated from ref. ^21^ based on a strain rate of 1 × 10^−5^ s^−1^, dislocation density of 1 × 10^12^ m^−2^ and temperatures in the range 1,200–1,600 °C. The approximate ranges of lattice resistances occurring in the lithosphere and asthenosphere were estimated from ref. ^21^ based on a strain rate of 1 × 10^−14^ s^−1^, a dislocation density of 1 × 10^10^ m^−2^ and temperatures in the range 800–1,400 °C. The box labelled ‘Avalanches’ indicates the approximate lattice resistance estimated for the asthenospheric mantle, with the upper length scale being the approximate grain size of coarse-grained rocks from the upper mantle. This grain size probably limits the maximum size of individual dislocation avalanches, but avalanches may exhibit spatiotemporal correlations extending to greater length scales, as indicated approximately by the arrow labelled ‘Correlation’. The dashed black line marks *R* = 1, separating regimes of dominantly mild and wild plasticity above and below, respectively.

Having demonstrated that olivine displays dislocation avalanches at room temperature, and that those avalanches can be considered in the wider framework of mild-to-wild plasticity, we can use that framework to predict the behaviour of olivine at higher temperatures and slower strain rates^6,21^. Notably, two recent analyses of independent experimental datasets collected at temperatures up to 1,300 °C indicate that lattice resistance at the temperatures and strain rates of the asthenosphere is orders of magnitude less than previously thought, and instead strength during steady-state flow is imparted almost entirely by long-range elastic dislocation interactions (that is, the term *Gb* above)^21,35^. Importantly, whereas the strength of dislocation interactions is relatively insensitive to temperature and strain rate, the lattice resistance decreases substantially with increasing temperature and decreasing strain rate, resulting in a progressive increase in the wildness of plasticity. Figure 4 presents predictions for *R* as a function of lattice resistance *τ* and system size *L*, where the fields dominated by mild and wild plasticity are approximately demarcated by the *R* = 1 line. Figure 4 also indicates the approximate loci of various experiments and regions of the upper mantle. The plasticity of the lithospheric upper mantle at temperatures of ~800 °C is predicted to appear mild at all but the shortest length scales due to high lattice resistance at low temperatures. However, plasticity in the asthenospheric upper mantle at temperatures of ~1,300 °C is predicted to be extremely wild due to low lattice resistance at high temperature and slow strain rates. Notably, plasticity should appear wild at length scales at least up to the grain size.

At the high homologous temperatures and slow strain rates of the asthenosphere, the behaviour of olivine may be analogous to that of water ice, which is the wild endmember of plasticity due to its low lattice resistance and general lack of short-range dislocation interactions^12^ (Fig. 3). Acoustic-emission monitoring of dislocation avalanches in polycrystalline ice has revealed that individual dislocation avalanches are confined within individual grains^12,13^. However, avalanches exhibit spatial and temporal correlations over distances greater than the grain size, indicating that avalanches can trigger cascades in nearby grains and thereby extend the effective length and time scales of the strain-rate perturbation^12–14^. It remains an open question as to how large these correlated dislocation avalanches may become in the asthenosphere, where the system size is large and long timescales are available to capture low-probability, extensive, correlated avalanche events.

Overall, these considerations suggest that the upper mantle probably exhibits a mild-to-wild transition with increasing depth and temperature from the lithosphere into the asthenosphere. The wild plasticity predicted at high temperatures is a striking departure from the current paradigm of slow continuous creep. The predictions in Fig. 4 suggest that the existence of this mild-to-wild transition in olivine is testable in laboratory creep experiments, which should span the transition if conducted over temperatures in the range 1,200–1,600 °C. In such tests, dislocation avalanches may be detectable as acoustic emissions, which are predicted to become more intermittent and pronounced with increasing temperature. Previous experiments on olivine under such conditions have not focused on resolving subtle displacement bursts, nor have they attempted to measure acoustic emissions in a systematic manner, so such measurements are an important target for future investigation. We also note that nanoindentation creep tests on halite^36^ exhibit transient periods of dramatically accelerated deformation that provide an example of instabilities during power-law dislocation creep at homologous temperatures higher than those of our experiments.

Our findings raise intriguing questions about the nature of plasticity and viscous flow of olivine in the upper mantle and of other minerals in other geological contexts. Mild continuous deformation of the asthenosphere at large length scales observed from Earth’s surface could represent an average over many avalanche events and intervening stasis. In such contexts, where bulk strain rates are low, perturbations that are detectable at Earth’s surface may be vanishingly few. However, intermittent deformation phenomena in the viscous regime, such as deep earthquakes^7,8^ and slow-slip events^9^, are observed in regions of elevated strain rate, such as deforming slabs and shear zones, and are typically considered to be dependent on triggering by perturbations in material properties or the stress state at smaller length scales. Considering that our nanoindentation tests indicate that dislocation avalanches occur in olivine at room temperature (Figs. 1 and 2), that the framework of mild-to-wild plasticity predicts that deformation should get wilder with depth in Earth (Fig. 4), and that dislocation avalanches generally exhibit spatiotemporal correlations that effectively extend the range of the instability^12–14^, it is plausible that dislocation avalanches may trigger or enhance such transient macroscopic phenomena. For instance, inherently unstable plasticity could enhance instabilities initiated by, for example, phase transformation or shear heating in the context of deep earthquakes^7,8^. Unstable plasticity in other minerals may also contribute to slow-slip events in crustal shear zones^9^. These considerations motivate future investigation of mild-to-wild plasticity in other minerals and the potential interactions among dislocation avalanches and other deformation processes.

## Methods

### Nanoindentation data acquisition and processing

The spherical nanoindentation tests in this study were originally reported in ref. ^26^, and further details and data are available therein. The methods used for acquiring and processing the data are also described here. Tests were performed using an MTS XP nanoindenter and a spheroconical diamond indenter tip. In all tests, the nominal strain rate (loading rate divided by load) was kept constant at 0.05 s^−1^.

Nanoindentation tests were first performed on a fused-silica standard to determine the effective radius of the tip. We performed 16 purely elastic indents with maximum displacements into the sample in the range of 40–70 nm. The effective modulus, *E*_eff_, of the indentation test, which takes into account the elasticity of both the sample and the indenter tip, was calculated as

S1 $$\frac{1}{{E}_{{\rm{eff}}}}=\frac{1-{\nu }_{{\rm{s}}}^{2}}{{E}_{{\rm{s}}}}+\frac{1-{\nu }_{{\rm{i}}}^{2}}{{E}_{{\rm{i}}}}$$

where *E* is Young’s modulus, *v* is Poisson’s ratio, and subscripts i and s refer to the indenter tip and the sample, respectively. The effective radius of the indenter tip, *R*_eff_, was then calculated using the equation for a Hertzian contact:

S2 $$P=\frac{4}{3}{E}_{{\rm{eff}}}{R}_{{\rm{eff}}}^{1/2}{h}^{3/2}$$

In this equation, *P* is load and *h* is displacement of the tip into the sample surface. As the indents in fused silica were purely elastic, *R*_eff_ is taken to be equal to the true indenter radius.

Nanoindentation experiments on olivine were carried out using a tip with an effective radius of 3 μm. After using the method of Kalidindi and Pathak^37^ to determine the effective touch point for each test, *E*_eff_ was calculated for each crystal by fitting the Hertzian contact relationship to purely elastic indents (70-nm deep) using the calibrated indenter radius.

We use Kalidindi and Pathak’s^37^ definitions of contact radius *a* and strain *ε*, as they produce curves that are good analogues of stress–strain curves. As our experiments were performed using continuous stiffness measurement, the resulting contact radius, hardness *H* and strain could be calculated at all points in the test as

S3 $$a=\frac{S}{2{E}_{{\rm{eff}}}}$$

S4 $$H=\frac{P}{\pi {a}^{2}}$$

and

S5 $$\varepsilon =\frac{4}{3\pi }\frac{h}{a}$$

The precision of load and displacement measurements on the MTS XP nanoindenter were 50 nN and <0.01 nm, respectively, according to the manufacturer’s specifications. However, the precision of a real measurement is driven as much by the laboratory environment as the machine specifications. A reasonable approximation of the precision can be estimated using a running s.d. and running average. Such estimates should be conservative, as all values of interest (load, displacement, stiffness, hardness and strain) generally increase throughout the experiment. The precision of the three machine-generated values (load, displacement and stiffness) was ~1–2%. The precision of hardness and strain were ~2–3% and 1–2%, respectively.

Surface-breaking fractures were visible around most indents after the experiments^23^. However, both theoretical considerations and observational evidence indicate that, when indented by a tip with a radius on the order of a few micrometres or less, only dislocation motion occurs during loading, whereas microcracks initiate during unloading, even in brittle ceramics^26,27,38^. Similarly, examination of the fracture structure using focused-ion-beam milling suggested that these fractures result from the unloading process as residual stresses are released^26^. These interpretations are supported by the similar log-normal forms of the distributions of secondary displacement bursts occurring during spherical nanoindentation of olivine (Fig. 2) and Cu^17^. As Cu does not fracture during nanoindentation with either spheroconical^28,29^ or sharp^30,31^ tips, this similarity provides further confirmation that secondary displacement bursts with these characteristics are formed by dislocation motion alone.

### Analysis of displacement bursts

We analysed displacement bursts in the plastic-flow portion of each test after the larger initial displacement burst of the primary pop-in. To automatically detect displacement bursts in the load–displacement curve, we detected peaks in the ratio of displacement to load as a function of displacement. To set the threshold for peak detection in the plastic-flow portion of each test, we analysed changes in the gradient of the load–displacement curve during the elastic-loading portion of the same test. We assumed that fluctuations in displacement/load during elastic loading arise from instrument noise, temperature variation and/or other factors unrelated to plasticity. We measured the prominences of peaks in the displacement/load series during the final 10 s of elastic loading before the primary pop-in, and took 3 s.d. of these peak prominences to be the threshold prominence for peak detection in the displacement/load series during the plastic-flow portion of the same test. In other words, displacement bursts were detected in the plastic-flow portion of a test if they were more pronounced than 99% of the fluctuations occurring during the latter stages of elastic loading. The detected displacement bursts were checked visually on each load–displacement curve (Fig. 1).

To estimate the plastic strain that occurred during each displacement burst, we analysed the indentation hardness–strain series during the plastic-flow portion of each test. To estimate differential stress during the indentation experiments, the hardness is often downscaled by a factor termed the constraint factor to account for the confining effect of the sample surrounding the deforming zone^39^. The value of this constraint factor ranges from 1 for a perfectly elastic material to 3 for a perfectly plastic material^39^. However, determination of the appropriate constraint factor to use in spherical nanoindentation is complex and poorly established^40,41^, especially for materials such as olivine that exhibit elastic anisotropy^42^, plastic anisotropy^6,26^ and kinematic hardening by the generation of back stress^6^. We thus took a simple and conservative approach of assuming a constraint factor of 1 and thereby corrected for elastic effects based on changes in hardness. In doing so, we maximized elastic–strain corrections to the total strain, resulting in conservative, lower-bound estimates of average plastic strain during displacement bursts and the wildness of each sample. To perform this correction for elastic effects, we used the Young’s modulus specific to each sample^26^. Furthermore, if the true duration of a displacement burst was less than the 0.2-s sampling interval, then the plastic strain during the displacement burst would be overestimated due to a contribution of steady plastic flow also occurring during the sampling interval. For a typical rate of steady plastic flow on the order of 1.5 × 10^−3^ s^−1^, a plastic strain of 3 × 10^−4^ would accrue in 0.2 s by steady plastic flow if the displacement burst was instantaneous. As the mean plastic strain occurring during the 0.2-s sampling intervals associated with the displacement bursts was 1.3 × 10^−3^, these strains might overestimate those actually occurring during the displacement bursts by up to ~20% (that is, the strain contributed instead by steady plastic flow) depending on the true durations of the displacement bursts.

## Online content

Any methods, additional references, Nature Portfolio reporting summaries, source data, extended data, supplementary information, acknowledgements, peer review information; details of author contributions and competing interests; and statements of data and code availability are available at 10.1038/s41561-026-01920-7.

## Data Availability

Data are available from Apollo, the University of Cambridge Repository^43^.

## Code availability

Code is available from Apollo, the University of Cambridge Repository^43^.
